# Hypotension-driven continuous watershed cerebral infarction secondary to critical coronary artery disease: A case report

**DOI:** 10.1097/MD.0000000000048436

**Published:** 2026-04-17

**Authors:** Huizhen Lu, Dejun Wu, Xiaoqiang Mao, Weifeng Jiang

**Affiliations:** a Department of Neurology, The Quzhou Affiliated Hospital of Wenzhou Medical University, Quzhou People’s Hospital, Quzhou, China; b Department of Gerontology, The Quzhou Affiliated Hospital of Wenzhou Medical University, Quzhou People’s Hospital, Quzhou, China.

**Keywords:** case report, coronary artery disease, heart–brain syndrome, hypotension, ischemic stroke, watershed infarction

## Abstract

**Rationale:**

Refractory hypotension may aggravate cerebral hypoperfusion in acute ischemic stroke, but occult coronary artery disease is often overlooked.

**Patient concerns:**

A 67-year-old man presented with sudden right-sided weakness. Within 24 hours, he developed persistent hypotension (approximately 90–100/50–60 mm Hg) and neurological worsening.

**Diagnoses:**

Brain magnetic resonance imaging revealed an acute left frontoparietal watershed infarction. Despite fluid resuscitation and vasopressors, hemodynamic instability persisted. Hypovolemia, endocrine disorders, and cervico-cephalic stenosis were excluded. Although cardiac biomarkers and ejection fraction were normal, electrocardiography showed inferior Q waves. Coronary angiography confirmed critical left anterior descending (LAD) artery stenosis with distal occlusion.

**Interventions:**

Fluid resuscitation and vasopressor support failed to stabilize blood pressure. Balloon angioplasty was performed for the left anterior descending artery lesion.

**Outcomes:**

After intervention, blood pressure normalized without vasopressors, and neurological function improved.

**Lessons:**

In acute ischemic stroke patients with unexplained refractory hypotension and watershed infarction, occult coronary ischemia should be considered even when troponin is normal. Early coronary evaluation and timely revascularization may improve cerebral perfusion and outcomes.

## 1. Introduction

Acute ischemic stroke (AIS) is highly sensitive to systemic hemodynamics.^[1]^ While guidelines emphasize permissive hypertension to optimize cerebral perfusion, hypotension in the acute phase is uncommon and clinically hazardous, predisposing to border‑zone (watershed) infarction and neurological deterioration by further reducing cerebral blood flow in regions with tenuous collateral supply.^[2-4]^ Recognizing and correcting the cause of hypotension early is therefore integral to secondary brain injury prevention in AIS.^[5,6]^

The etiologies of hypotension in the stroke setting are diverse and often multifactorial, including hypovolemia, medication effects, autonomic dysregulation, endocrine disorders, sepsis, and cardiogenic causes.^[6]^ Importantly, acute coronary syndromes (ACS) and occult critical coronary artery disease can coexist with, precipitate, or be unmasked by AIS through the bidirectional “stroke–heart” interaction.^[7,8]^ Such cardiac involvement may be clinically silent or atypical presenting without chest pain and with non‑diagnostic biomarkers, yet still produce a low‑flow state sufficient to trigger hemodynamic cerebral ischemia.^[7]^ Inferior Q waves on electrocardiography, even with preserved ejection fraction, should prompt consideration of prior myocardial infarction and coronary ischemia as contributors to unexplained hypotension.

This case describes an older adult with an acute left frontoparietal watershed infarct who developed refractory hypotension and rapid neurological worsening despite guideline‑directed stroke therapy and the absence of significant cervico‑cephalic stenosis. Systematic evaluation uncovered severe left anterior descending coronary disease with distal occlusion, providing a plausible low‑flow substrate for the hemodynamic stroke and the patient’s instability.

## 2. Case presentation

**Figure 1.**
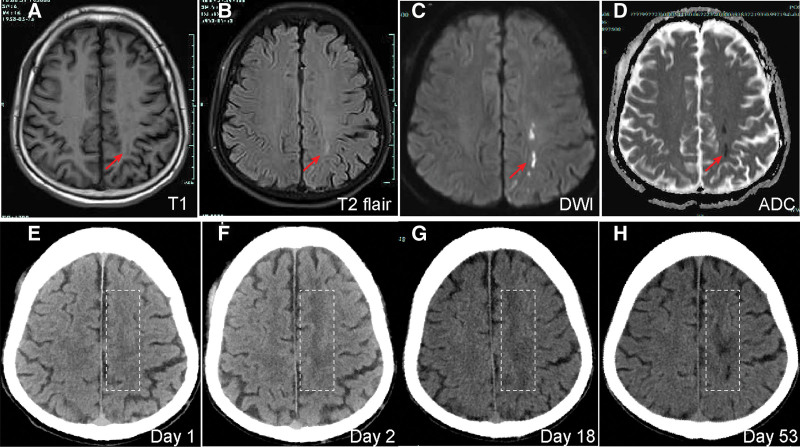
Multimodal imaging and longitudinal evolution of the watershed infarction. (A–D) Admission MRI (day 1): T1-weighted (A) and T2-FLAIR (B) images show subtle changes, while DWI (C) and ADC (D) confirm acute restricted diffusion in the left frontoparietal watershed zone. (E–H) Sequential cranial CT images obtained on day 1 (E), day 2 (F), day 18 (G), and day 53 (H) demonstrate the progression of the lesion from the acute phase to chronic encephalomalacia. The red arrows and the white boxes indicate the lesion in the left parietal lobe and its evolution. ADC = apparent diffusion coefficient, CT = computed tomography, DWI = diffusion-weighted imaging, MRI = magnetic resonance imaging.

A 67-year-old man presented with a 1-day history of sudden right-sided limb weakness. He had no history of hypertension, diabetes, or dyslipidemia, but reported long-term smoking and alcohol use. On admission, neurological examination revealed clear consciousness without aphasia or cognitive impairment. Cranial nerve examination was unremarkable. Motor examination showed weakness in the right upper and lower extremities (grade 4/5). Sensory examination indicated intact sensation. Deep tendon reflexes were symmetric, and the Babinski sign was positive on the right side. The NIHSS score was 2, and the modified Rankin Scale (mRS) score was 3. Brain magnetic resonance imaging (MRI) performed on admission (day 1) revealed acute ischemic infarction (Fig. 1A–D). Specifically, diffusion-weighted imaging (DWI) showed a high-signal pattern confined to the left internal watershed zone, accompanied by a low signal on the apparent diffusion coefficient (ADC) map (Fig. 1A–D). T1-weighted and T2-FLAIR sequences showed subtle signal changes consistent with the hyperacute phase (Fig. 1A–D). Standard secondary prevention was initiated with enteric-coated aspirin 100 mg once daily, clopidogrel 75 mg once daily, and atorvastatin 40 mg once daily, together with intravenous fluid support.

Within 24 hours, continuous hemodynamic monitoring documented refractory hypotension with systolic blood pressure of 90 to 100 mm Hg and diastolic blood pressure of 50 to 60 mm Hg. Despite volume resuscitation and a metaraminol bitartrate infusion, neurological deficits did not improve and rehabilitation was limited due to hemodynamic instability. The patient experienced neurological worsening, with right lower limb strength declining to 0/5 and the NIHSS increasing to 6. Oral antiplatelet agents were temporarily withheld and intravenous tirofiban was initiated. To monitor the evolution of the infarction, sequential non-contrast CT scans were performed. While the day 1 CT was unremarkable, follow-up scans on day 2 and day 18 revealed progressive hypodensity in the left frontoparietal region. A repeat CT on day 53 confirmed a well-defined area of encephalomalacia, consistent with the permanent sequelae of the watershed infarction (Fig. 1E–H).

Cardiac evaluation was performed to investigate the etiology of the hypotension (Table 1). Serial monitoring of cardiac biomarkers was conducted. While a transient, mild elevation in CK-MB (peak 34.9 U/L on day 18; reference: 5–25 U/L) was observed, high-sensitivity Troponin I (hs-TnI) remained consistently within the normal range (<0.002 μg/L; reference: <0.026 μg/L) at all time points (days 1, 18, 24, and 53). Similarly, B-type natriuretic peptide (BNP) levels remained low (<20 pg/mL; reference: <100 pg/mL). Standard 12-lead ECGs performed on days 1, 10, 15, 18, and 24 consistently revealed pathological Q-waves in the inferior leads (II, III, aVF). No dynamic ST-segment elevation was observed. By day 53, the follow-up ECG demonstrated stable sinus rhythm. Transthoracic echocardiography demonstrated a left ventricular ejection fraction of 67%. An endocrine evaluation, including thyroid function tests, cortisol, adrenocorticotropic hormone, and pituitary profile, was unremarkable. Carotid duplex ultrasound and intracranial magnetic resonance angiography revealed no significant extracranial or intracranial stenosis or occlusion. The infarct exhibited a watershed distribution, compatible with hypoperfusion.

**Table 1:** Serial evolution of cardiac biomarkers.

Time point	CK-MB (U/L)	hs-TnI (μg/L)	BNP (pg/mL)
Reference range	5–25	<0.026	<100
Day 1	21.2	0.001	<5.0
Day 18	34.9	0.001	<10.0
Day 24	25.6	0.002	13.3

BNP = B-type natriuretic peptide, CK-MB = creatine kinase-myocardial band; hs-TnI, high-sensitivity troponin I.

**Figure 2.**
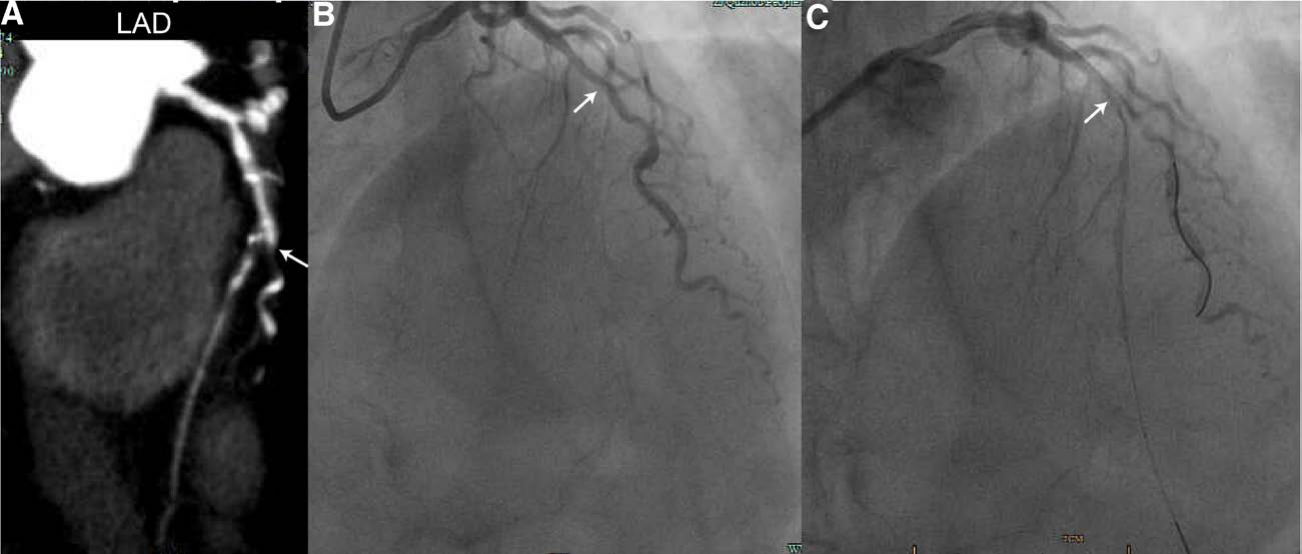
(A) Coronary CT angiography demonstrates mixed and noncalcified plaques in the LAD with mild-to-severe luminal stenoses across segments. (B) Digital subtraction angiography (DSA) shows approximately 60% ostial LAD stenosis and complete occlusion distal to the proximal–mid segment. (C) Post-balloon angiogram reveals residual moderate-to-severe proximal stenosis and severe stenosis near the second diagonal branch origin. CT = computed tomography, DSA = digital subtraction angiography, LAD = left anterior descending.

On day 24, coronary angiography was performed to investigate the etiology of the hemodynamic instability. The study revealed severe multivessel disease (Fig. 2). The left main coronary artery showed 30% stenosis; the left anterior descending (LAD) artery presented with 60% ostial stenosis and total occlusion in the mid-proximal segment; the left circumflex (LCX) and dominant right coronary artery (RCA) showed diffuse stenosis (60–90%). Percutaneous coronary intervention (PCI) was initiated for the LAD lesion. A 6F EBU 3.5 guiding catheter was engaged, and a Gaia 3 guidewire successfully crossed the occluded segment. Pre-dilation was performed sequentially using Sprinter 1.5 mm × 15 mm and 2.0 mm × 20 mm balloons to the mid-distal and proximal segments. Although follow-up angiography showed restored antegrade flow (TIMI 3), a residual 60% to 70% stenosis persisted in the proximal LAD, and a dissection was suspected distally. Intravascular ultrasound (IVUS) was performed to guide decision-making. IVUS confirmed severe plaque burden (85%) with a minimum lumen area (MLA) of 1.76 mm^2^ in the mid-segment. Crucially, it identified a myocardial bridge in the mid-distal segment (causing 60% systolic compression) and an intramural hematoma distal to the bridge. Given the high risk of determining stent sizing and the danger of propagating the hematoma or perforating the bridged segment, stenting was deferred. The procedure was concluded with the successful restoration of TIMI 3 flow in the LAD.

The long-term management strategy focused on secondary prevention and monitoring of the coronary anatomy. Since stenting was deferred, the patient was prescribed dual antiplatelet therapy (aspirin 100 mg and clopidogrel 75 mg daily) for 3 months to mitigate the risk of thrombosis at the site of the residual dissection and severe atherosclerosis. This was accompanied by lipid-lowering therapy (rosuvastatin 10 mg daily) to stabilize plaque burden. Strict blood pressure monitoring was emphasized to balance cerebral perfusion with cardiac workload. A follow-up coronary angiography was scheduled for 3 months later to evaluate the healing of the intramural hematoma and the progression of the residual stenosis in the LAD.

**Figure 3.**
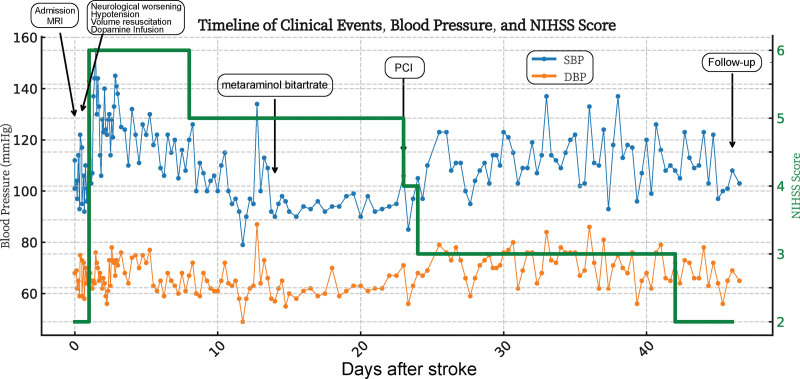
Timeline of clinical events, diagnostic findings, and therapeutic interventions. The chart illustrates the correlation between the refractory hypotension episodes, the progression of neurological symptoms (NIHSS score), and the normalization of hemodynamic status following percutaneous coronary intervention (PCI). DBP = diastolic blood pressure, MRI = magnetic resonance imaging, NIHSS = National Institutes of Health Stroke Scale, PCI = percutaneous coronary intervention, SBP = systolic blood pressure.

Approximately 6 weeks after the stroke, systemic blood pressure increased steadily and vasopressors were discontinued, with blood pressure stabilizing around 112/73 mm Hg. With rehabilitation, neurological deficits improved; at the most recent assessment, the NIHSS had decreased to 2 and the mRS was 3. The comprehensive timeline of the clinical course, including blood pressure trends, NIHSS scores, and key interventions, is summarized in Figure 3.

## 3. Patient perspective

“When I first felt the dizziness and weakness, I was terrified, especially when the doctors told me my blood pressure kept dropping despite treatment. I felt extremely fatigued and lightheaded, like I was going to pass out constantly. I didn’t feel chest pain, so I was surprised when they found a problem in my heart. After the heart procedure, I felt my energy return almost immediately. I am now able to walk independently and am grateful that the doctors found the root cause in my heart, not just treating my brain.”

## 4. Discussion

This case highlights the clinical significance of persistent hypotension in AIS and its propensity to drive a hemodynamic pattern of injury. The patient developed early, refractory blood pressure instability accompanied by neurological worsening and an MRI pattern consistent with border‑zone infarction. In the absence of significant extracranial or intracranial stenosis, this constellation strongly supports reduced systemic perfusion as the dominant mechanism of cerebral ischemia rather than artery‑to‑artery or cardioembolic occlusion. Given that border‑zone territories are especially vulnerable when autoregulatory capacity is impaired, even modest decrements in mean arterial pressure can convert penumbra to core; timely identification and correction of systemic contributors therefore become central to secondary brain injury prevention.

The subsequent cardiac evaluation provides an instructive example of the stroke–heart interface. Although cardiac biomarkers were within reference limits and left ventricular ejection fraction was preserved, the electrocardiographic inferior Q waves raised pretest probability for prior myocardial injury. Coronary imaging then revealed severe left anterior descending disease with distal occlusion, offering a biologically plausible substrate for impaired circulatory reserve during the acute neurovascular illness. Preserved resting ejection fraction does not exclude limited capacity to augment stroke volume under stress, and clinically important coronary disease may present without biomarker elevation. In this context, the identification of significant coronary pathology in a patient with otherwise unexplained hypotension aligns with the observed watershed cerebral injury and supports a low‑flow pathophysiological model, even though strict causality cannot be proven.

Management in this setting requires navigating competing hemodynamic priorities. Permissive hypertension is generally favored in AIS to preserve collateral perfusion.^[9]^ However, when spontaneous blood pressure is inadequate, short‑acting vasopressor support and cautious volume optimization become necessary to secure a stable mean arterial pressure without precipitating pulmonary congestion.^[10]^ In this case, despite fluid resuscitation and vasopressor therapy, hypotension initially persisted and constrained rehabilitation efforts, underscoring how systemic instability can amplify neurological morbidity. The temporary transition from oral dual antiplatelet therapy to intravenous tirofiban provided continuous platelet inhibition during a period of hemodynamic fragility and anticipated coronary procedures, while retaining the ability to titrate rapidly around invasive interventions and neuroimaging reassessments. Coronary angiography confirmed severe LAD involvement, and balloon angioplasty was attempted but terminated in the setting of residual stenoses and distal dissection. The subsequent steady rise in systemic blood pressure and successful weaning from vasopressors, coupled with neurological improvement on rehabilitation, suggest evolving hemodynamic stabilization, although the relative contributions of coronary manipulation, autonomic recovery after stroke, and natural course cannot be disentangled from available data.

The diagnosis of hypotension-driven stroke in this case relied on the rigorous exclusion of alternative etiologies. Hypovolemic shock and occult hemorrhage were ruled out given the patient’s lack of fluid loss history, stable hemoglobin levels, absence of clinical bleeding signs, and nonresponse to aggressive fluid resuscitation. Septic shock was excluded based on normal inflammatory markers (WBC, procalcitonin) and the absence of fever. We also considered adrenal insufficiency but ruled it out following normal serum cortisol and ACTH findings. Furthermore, medication-induced hypotension was excluded as the patient was not prescribed antihypertensives or other vasoactive agents prior to admission. Primary autonomic dysfunction was deemed unlikely due to the acute onset of hypotension, the absence of a prior history of orthostatic intolerance, and the lack of concurrent autonomic symptoms such as anhidrosis. Crucially, we further distinguished this case from stroke-induced autonomic dysfunction. Literature indicates that acute ischemic stroke, particularly involving the central autonomic network (e.g., insular cortex, hypothalamus, or brainstem), can disrupt sympathetic outflow, leading to hemodynamic instability or autonomic symptoms such as hyperhidrosis.^[11]^ This distinct temporal correlation confirms that the hypotension was cardiogenic (“pump failure” due to silent ischemia) and the primary driver, rather than a consequence, of the cerebral infarction. Ultimately, the diagnosis of cardiogenic hypotension secondary to critical coronary ischemia was solidified by the distinct temporal correlation: the patient’s blood pressure remained refractory to conventional pressors but normalized immediately following the revascularization of the LAD stenosis.

Our case makes a specific contribution to the literature on the “heart–brain axis” by distinguishing hemodynamic compromise from the more commonly reported cardioembolic mechanisms. While current guidelines emphasize screening for atrial fibrillation to prevent embolic stroke, this case underscores that cardiac “pump failure” due to critical coronary stenosis can directly cause cerebral hypoperfusion and watershed infarction. The uniqueness of this case lies in the patient’s presentation: the refractory hypotension was the primary indicator of severe coronary artery disease, occurring without chest pain (silent ischemia). This highlights a critical clinical pearl: in the setting of watershed infarcts, the heart should be viewed not just as a source of emboli, but as a dynamic pump whose failure can drive cerebral ischemia.

This case adds several practical considerations for stroke units confronted with refractory hypotension. First, a structured search for reversible systemic causes should extend to targeted cardiac work‑up even when troponin is normal and ejection fraction appears preserved, particularly in the presence of suggestive electrocardiographic abnormalities or a watershed infarct pattern. Second, early, active optimization of blood pressure to protect vulnerable border‑zone tissue is critical and should be coordinated with cardiology to balance cerebral perfusion goals against myocardial demand. Third, antithrombotic strategies and the timing of coronary intervention require individualized, multidisciplinary deliberation to reconcile ischemic protection with hemorrhagic risk in the post‑stroke period. Overall, the case underscores the importance of integrating neurovascular and coronary assessments when AIS is complicated by persistent hypotension.

## 5. Limitations

This is a single-case report, so causal inference is limited. We cannot fully exclude the contribution of autonomic dysfunction, subtle cardiac output changes, or other unrecognized systemic factors. In addition, long-term follow-up angiography was not yet available at the time of manuscript preparation, and the durability of coronary patency therefore remains to be confirmed.

## 6. Future directions

Future studies should investigate how often occult coronary ischemia contributes to refractory hypotension in AIS, especially in patients with watershed infarction. Prospective multicenter studies are needed to determine whether early coronary evaluation, serial hemodynamic monitoring, and multidisciplinary stroke–cardiology management improve outcomes in this subgroup.

## 7. Conclusion

This case demonstrates that refractory hypotension can be the presenting manifestation of critical coronary artery disease in stroke patients and may lead to continuous watershed infarction. In AIS patients with unexplained, persistent hypotension and MRI findings consistent with hemodynamic failure, clinicians should maintain a high index of suspicion for occult cardiac ischemia even when chest pain is absent and troponin is normal. Early coronary evaluation and timely revascularization may restore cerebral perfusion and improve neurological outcomes.

## Acknowledgments

We would like to thank the patient and his family for their cooperation and permission to publish this case report.

## Author contributions

**Funding acquisition:** Weifeng Jiang, Xiaoqiang Mao.

**Investigation:** Huizhen Lu, Dejun Wu, Xiaoqiang Mao, Weifeng Jiang.

**Methodology:** Huizhen Lu, Dejun Wu, Xiaoqiang Mao, Weifeng Jiang.

**Project administration:** Weifeng Jiang.

**Resources:** Dejun Wu, Xiaoqiang Mao, Weifeng Jiang.

**Supervision:** Weifeng Jiang.

**Writing – original draft:** Huizhen Lu.

**Writing – review & editing:** Dejun Wu, Weifeng Jiang.
